# Perianal Paget disease treated with wide excision and thigh skin flap reconstruction: a case report and review of literature

**DOI:** 10.1097/MD.0000000000011638

**Published:** 2018-07-27

**Authors:** Kexin Shen, Hai Luo, Jun Hu, Zhongshi Xie

**Affiliations:** Department of Gastrointestinal Colorectal and Anal Surgery, China-Japan Union Hospital of Jilin University, Jilin, P.R. China.

**Keywords:** perianal Paget disease, surgery, thigh flap reconstruction

## Abstract

**Rationale:**

Extramammary Peget disease (EMPD) is a rare tumor, which typically occurs in the perianal regions. Perianal Paget disease (PPD) was first reported in 1893, and which has only 180 cases that have been reported in literature. The rarity of the disease means that no large studies have been made, and so the optimal treatment for this disease still remains controversial.

**Patient concerns:**

In this case, we reported a 65-years-old female patient with PPD. The patient suffered intermittent pruritus in the perianal region for 1 year. She had neither genitourinary nor gastrointestinal symptoms. Local examination revealed a whitish gray skin lesion in the left perianal area with a 3 × 3 cm size.

**Diagnoses:**

The perianal skin biopsy was consistent with EMPD. Then the patient underwent a screening colonoscopy, gynecological ultrasonography, and whole-body computed tomography to exclude underlying malignancy.

**Interventions:**

The patient underwent wide local excision with margin control by frozen section examination and posterior thigh flap reconstruction. Subsequently follow-up remains 6 years.

**Outcomes:**

The operation was successful. The total operation time was 296 minutes, and the estimated blood loss was 120 mL. The patient recovered without any complication and discharged home on the sixth postoperative day. After 6 years’ follow-up, there was no evidence of recurrence and no influence in the anal bowel control function.

**Lessons:**

PPD is a rare disorder; the current knowledge on diagnose and treatment is based on small case series. Thus, it is complicated to elaborate a consensus on diagnostic and treatment guidelines. Wide local excision remains the treatment of choice with a variety of adjuvant therapies. Our method has an advantage which is the posterior thigh flap could be designed in accordance with the defect of the perianal. It is mandatory that the patient must accept a long-term follow-up to detect local recurrence and to distant carcinoma.

## 1 Introduction

Extramammary Paget disease (EMPD) is a rare tumor that typically occurs in the perianal regions.^[1]^ Perianal Paget disease (PPD) was first reported by Darier and Couillaud in 1893.^[2]^ It has only 180 cases reported in literature, and so it is difficult to estimate the real incidence of PPDs.^[3]^

PPD is most often detected in old patients with similar morbidity in males and females.^[3]^ The clinical presentation of PPDs is different from mild rash or roseola. Perianal itchiness and pain are the most common chief complaints, and other symptoms include bleeding, mucous seepage, lump, and difficulty in defecating.^[4]^

The diagnosis is made by pathohistology through the presence of specific tumor cells called Paget cells. The rarity of the disease means that no large studies have been done, and so the optimal treatment of this disease still exits controversial. It is generally recommended for the treatment of noninvasive disease that is wide local excision of the skin and subcutaneous tissue in the perianal region, and various techniques are described in the literature for the reconstruction of these defects.

In the present report, we adopted the procedure that is wide local excision with margin control by frozen section examination. We performed a reconstruction technique to use a posterior thigh flap after resection of the perianal area.

## 2 Case report

A 65-year-old female came to our hospital in 2011 with history of intermittent pruritus in the perianal region for 1 year. The patient had neither genitourinary nor gastrointestinal symptoms, such as rectal bleeding, change in the bowel function, hematuria, dysuria, urinary frequency, or weight loss. Her family history was negative for skin, colorectal, or genitourinary cancer.

**Figure 1:**
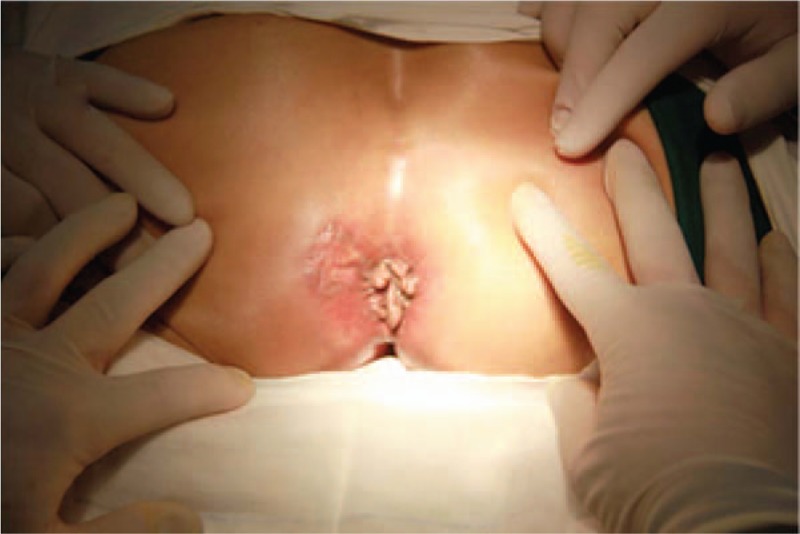
Left perianal area measureing 3 × 3 cm with surrounding lichenification.

Local examination revealed a whitish gray skin lesion in the left perianal area with a 3 × 3 cm size. The surrounding skin was lichenification (Fig. 1). Rectal examination was normal apart from mixed hemorrhoid. No enlarged inguinal lymph nodes were detected.

The perianal skin biopsy was consistent with EMPD. To exclude underlying malignancy, the patient was advised to undergo a screening colonoscopy, gynecological ultrasonography, and whole-body computed tomography (CT), which revealed unremarkable results. All laboratory examinations, including carcinoembrionic antigen, were normal.

**Figure 2:**
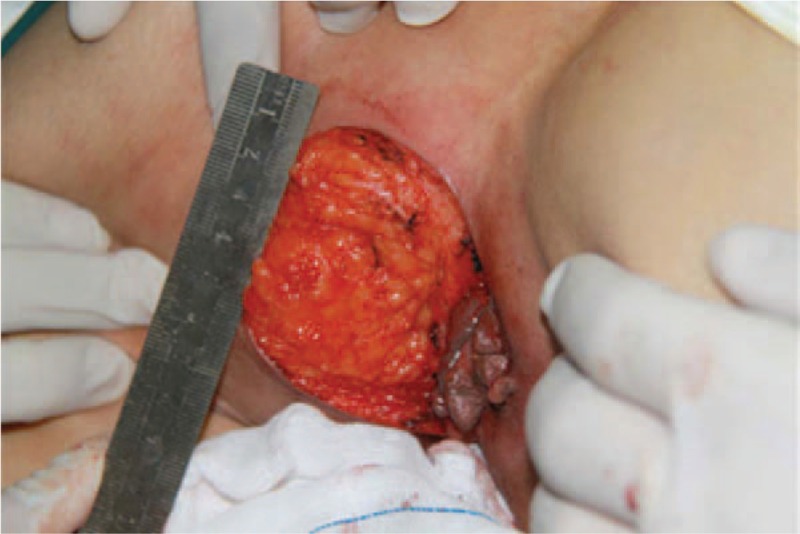
Defect of 7 × 6 cm in size.

**Figure 3:**
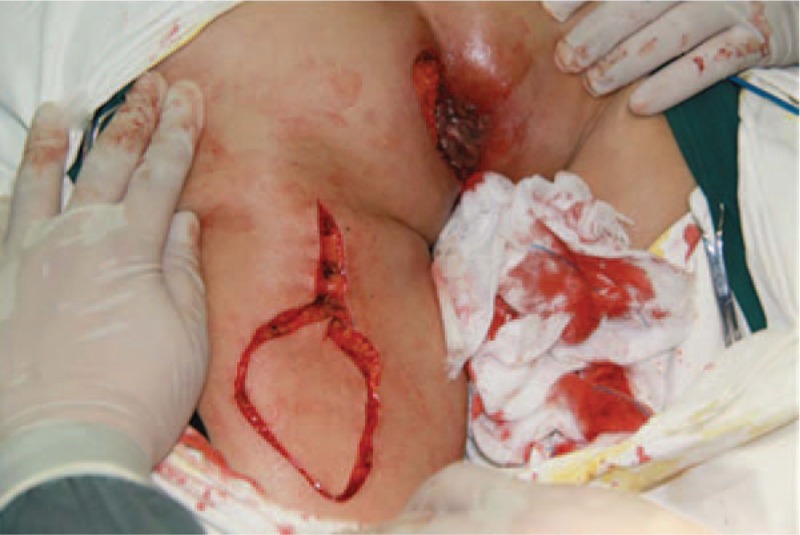
Posterior thigh flap designed according to the defect of perianal region.

**Figure 4:**
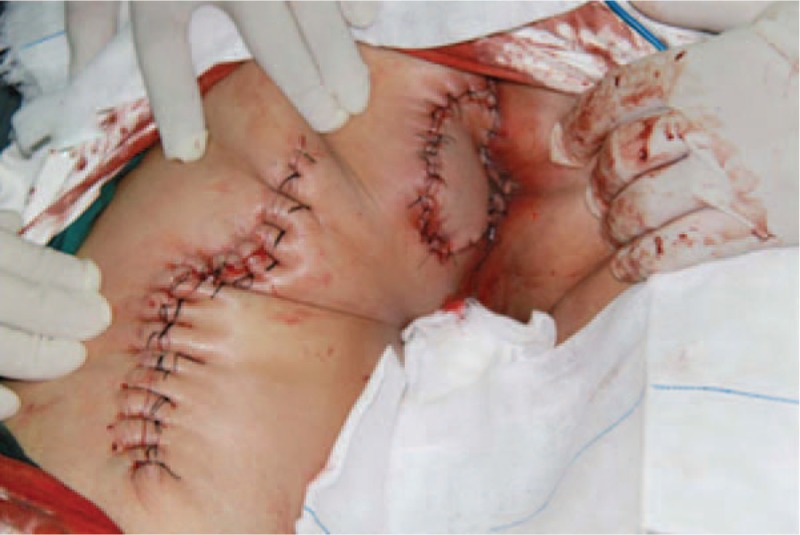
The defect was reconstructed with flap.

Informed consent was obtained from the patient for publication of this case report and accompanying images. After signing the informed consent, the patient was placed in a jack-knife position on the operation table after anesthesia. The operation consisted of a wide excision with frozen section control of the margins and a flap reconstruction. The perianal diseased skin and anal mucosa up to dentate line were integrally excised, preserving the external and internal sphincters. The size of the defect was 7 × 6 cm (Fig. 2). For the skin and the soft tissue defects, posterior thigh flap transposition was performed (Figs. 3 and 4). Then the dentate line was repaired and reinforced. A 28-Fr rectal tube was inserted into the anal canal. The total operation time was 296 minutes, and the estimated blood loss was 120 mL. The patient recovered without any complications and discharged home on the sixth postoperative day. After 6 years of follow-up, which included physical examination, ultrasonography, colonoscopy, CT scan, and random biopsies of the perianal skin, there was no evidence of recurrence and no influence in the anal bowel control function.

## 3 Discussion

PPD is an uncommon intraepithelial adenocarcinoma. The etiology of this disease is still not clear for now. Clinical feature is often nonspecific and insidious; thus, the diagnosis is frequently ignored. Patients commonly have symptoms like itching, irritation, rash, and pain, and bleeding may occur in long-term cases. These lesions typically appear in red or gray-white discoid dry bulge, and may turn into eczema or ulceration. The diagnosis could be determined through histological examination. The presence of Paget cells in the epidermis is direct evidence. Furthermore, CK7, CK19, and C-erb B2 are favorable immunohistochemical markers for the diagnosis of EMPD.^[5,6]^ Paget cells have the potential to invade and metastasize. The anatomic site of the PPD is strongly related to the underlying visceral carcinoma in 86% of cases.^[7]^ Our patients only have a skin lesion without associated colorectal carcinoma.

Treatment of PPD remains challenging and complicated by the nonspecific clinical situation and insidious onset of the disease. There is no standardized therapeutic guideline that we could choose. Myriad treatment options have been proposed for PPD, such as radiotherapy, chemoradiotherapy, CO_2_ laser ablation, imiquimod 5% cream and photodynamic therapy with topical aminolevulinic acid, but not enough data is currently available to define the role of noninvasive treatment.^[8]^ So, despite several controversies concerning PPD optimal therapeutic management, wide local excision of the skin and subcutaneous tissue in the perianal region is commonly adopted for the treatment of the disease. Spread of Paget cell could extend within the epidermis horizontally beyond clinical apparent disease.

Adequate microscopically clear margins are important to avoid clinical recurrence.^[9]^ As in our patient, on the edge of the defect, 6 points were taken for frozen-section analysis. However, wide local excision represents an aggressive operative management and usually results in a significant tissue defect which could not be covered up primarily without tension. Thus, it always causes remarkable morbidity and discomfort, and requires a special technique for its coverage.^[10]^ In most cases, primary suture or skin graft may not be suitable; the defect requires a flap transposition surgery. Moreover, reconstruction of perianal defects requires both the functional preservation and satisfactory cosmetic outcomes. According to the report, there are many techniques of flap transposition for covering the tissue defect areas, which have obtained satisfied outcomes, such as bilateral myocutaneous flaps of the gluteal or thigh muscles and V-Y island flaps and so on.^[11]^ The thigh offers great flexibility for the design of perforator flaps.^[12]^ Kishi et al^[13]^ reported the perianal reconstruction using a posterior thigh flap in 2010. They reported a satisfied outcome with this technique. In this case, we designed a posterior thigh perforator flap to cover the defect. The posterior thigh flap has shown advantages in the reconstruction of perianal defects compared to other flaps, such as the appropriate distance from the donor site to the defects, which makes the transposition easier. Furthermore, the well-supplied flap significantly increases the survival rate of the transplanted flap.^[14]^

Common complications include flap or graft failure, stricture and anal ectropion after local flap or skin graft. According to the extent of PPD, a colostomy may be required before wide local excision. Fecal diversion has been recommended by Shutze and Gleysteen,^[15]^ when more than half of the perianal region have to be removed, or the radius of the excision is >3 cm. In this case, the patients were refused to do colostomy, so we inserted a 28-Fr rectal tube into the anal canal to diverse the fetal.

Long-term follow-up of PPD patients is very critical. The focus is on monitoring disease recurrence and development of an associated cancer.^[16]^ The margin of the old perianal lesion should be examined by punch biopsy every year, and the patient should be referred to colonoscopy examination every 2 years during the follow-up period.^[17]^

## 4 Conclusion

PPD is a rare disorder. The current knowledge on diagnosis and treatment is based on small case series, which makes it complicated to elaborate a consensus on diagnosis and treatment guideline. Wide local excision remains the treatment of choice with a various adjuvant therapies. Our methods have the advantage that posterior thigh flap can be designed in accordance with the defect of the perianal. Long-term follow-up for detecting local recurrence and distant carcinoma is mandatory.

## Author contributions

K.S. participated in surgery, collected clinical information, and did the written work. H.L. and J.H. participated in surgery and collected the clinical data. Z.X. proposed and designed the procedure, and was the main operator in surgery.

**Conceptualization:** Hai Luo.

**Methodology:** Zhongshi Xie.

**Writing – original draft:** Hai Luo, Jun Hu.

**Writing – review & editing:** Kexin Shen.
